# THE NATIONAL ELDER MISTREATMENT STUDY 8 YEARS LATER: VICTIM MENTAL HEALTH OUTCOMES AND PROTECTIVE FACTORS

**DOI:** 10.1093/geroni/igz038.1773

**Published:** 2019-11-08

**Authors:** Melba Hernandez-Tejada, Ron Acierno, Georgia Anetzberger, Daniel Loew, Wendy Muzzy

**Affiliations:** 1 Medical University of South Carolina, Charleston, South Carolina, United States; 2 Case Western Reserve University, Cleveland, Ohio, United States; 3 Abt SRBI, New York, New York, United States

## Abstract

The purpose of this study was to conduct an 8-year follow-up of the National Elder Mistreatment Study (NEMS) and specify risk ratios for negative outcomes of elder abuse, including DSM-5 defined depression, generalized anxiety disorder (GAD), post-traumatic stress disorder (PTSD), and poor self-reported health. Methodology: Attempts were made to re-contact, via Computer Assisted Telephone Interview, all 752 NEMS participants who reported mistreatment since age 60 at Wave I, as well as a randomly selected sample of non-mistreated NEMS participants Results: 183 NEMS Wave I elder abuse victims and 591 non-victims provided data. In bivariate analyses, elder mistreatment 8 years earlier increased risk of negative outcomes by 200-700%. However, multivariate analyses revealed that Current (Wave II) social support was highly protective against most negative outcomes (excepting PTSD), and even appeared to nullify effects of mistreatment on GAD and poor self-reported health. Conclusions: Outcomes of elder mistreatment have not been studied prospectively in a national sample. The NEMS 8-year follow-up findings indicate a strong relationship between elder mistreatment at Wave I and negative emotional and physical health 8 years later. Fortunately, current (Wave II) social support appears to be both consistently and powerfully protective against most negative outcomes.

